# Supplementary material for: Zinc-Dependent Histone Deacetylases in Lung Endothelial Pathobiology
Source: Biomolecules. 2024 Jan 23;14(2):140. doi: 10.3390/biom14020140 (PMC10886568; doi:10.3390/biom14020140)
Supplement: Supplementary file 1 [file biomolecules-14-00140-s001.zip › biomolecules-2827905-supplementary.pdf]

Table S1: Molecular and structural characteristics of zinc-dependent HDACs

Isoform	PDB structure code	UniProtKB accession	Location on chromosome	Molecular weight (kDa)
HDAC1	4BKX_2	Q13547	1p35.2-p35.1	55.103
HDAC2	3MAX_1	Q92769	6q21	55.364
HDAC3	4A69_1	O15379	5q31.3	48.848
HDAC4	2VQV_1	P56524	2q37.3	119.040
HDAC5	AF_AFQ9UQL6F1_1	Q9UQL6	17q21.31	121.978
HDAC6	AF_AFQ9UBN7F1_1	Q9UBN7	Xp11.23	131.419
HDAC7	3C0Y_1	Q8WUI4	12q13.11	102.927
HDAC8	1T64_1	Q9BY41	Xq13.1	41.758
HDAC9	AF-Q9UKV0-F1	Q9UKV0	7p21.1	111.297
HDAC10	AF-Q969S8-F1	Q969S8	22q13.33	71.445
HDAC11	AF-Q96DB2-F1	Q96DB2	3p25.1	39.183

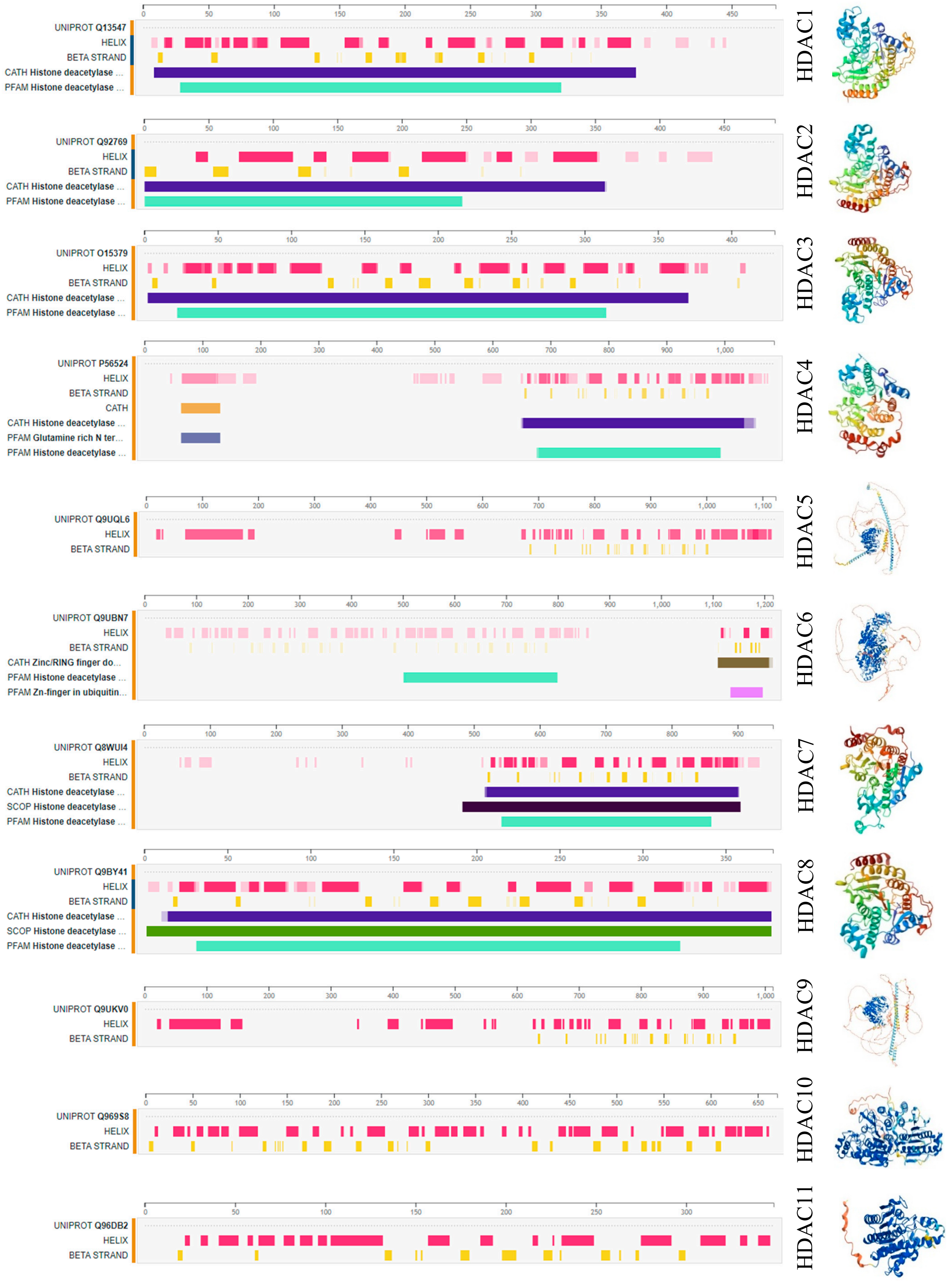

Figure S1: Secondary and tertiary structures of zinc-dependent HDACs derived upon protein data bank and UniProt.
